# Epidemiology of cutaneous leishmaniasis in Karachi, Pakistan

**DOI:** 10.1016/j.jdin.2025.03.002

**Published:** 2025-04-09

**Authors:** Farhan Mir Shaikh, Sandesh Raja, Azzam Ali, Adarsh Raja, Iftikhar Athar Rasool, Arfa Asad, Muhammad Sohaib Asghar

**Affiliations:** a Consultant Dermatologist, Department of Dermatology, Institute of Skin Diseases Sindh, Karachi, Pakistan; b Department of Dermatology, Dow Medical College, Dow University of Health Sciences, Karachi, Pakistan; c Department of Dermatology, Shaheed Mohtarma Benazir Bhutto Medical College Lyari, Karachi, Pakistan; d Department of Dermatology, Mohtarma Shaheed Benazir Bhutto General Hospital, Quetta, Pakistan; e Department of Internal Medicine, AdventHealth Sebring, Sebring, Florida

**Keywords:** cutaneous leishmaniasis, epidemiology, Karachi, public health, vector-borne disease

## Abstract

**Introduction:**

Cutaneous leishmaniasis (CL), caused by Leishmania species is a prominent neglected tropical disease globally, posing substantial public health challenges. This study explores the epidemiology, clinical characteristics, geographic distribution of CL cases, and role of travel to endemic areas contributing spread of the disease to urban centers like Karachi.

**Methods:**

A retrospective study was conducted from July 2019 to February 2024, enrolling 525 patients with confirmed CL diagnoses at a leading dermatological hospital in Karachi. Demographic and clinical data, including age, gender, lesion characteristics, and travel history were collected. The chi-square test was used to compare travel history to endemic areas between individuals from Karachi and those from other regions.

**Results:**

CL predominantly affected younger age groups, with 35.5% of cases occurring in individuals aged ≤10 years. Male patients accounted for 65.1% of the cohort. Lesions were predominantly located on exposed body parts (96.3%). Most patients in this study were from Baluchistan (49.7%). In Karachi, 37% of patients reported a travel history to endemic areas, whereas only 2.17% of cases from other regions had traveled (*P* < .001).

**Conclusion:**

The findings underscore the need for region-specific prevention and control strategies, enhanced surveillance, and public health initiatives to mitigate the spread of CL.

**Capsule Summary**

• Cutaneous leishmaniasis (CL) is a prominent neglected tropical disease globally and prevalent in rural areas of Pakistan.
• Forty-nine percent of the cohort belonged to Baluchistan and most patients report a travel history to endemic areas (37%). The findings underscore the need for region-specific prevention control strategies especially in urban nonendemic areas.

## Introduction

Cutaneous leishmaniasis (CL), ranked among the top 10 neglected tropical diseases, is a vector-borne infection spread by female sandflies. According to the World Health Organization (WHO), over 1 billion individuals inhabit areas where leishmaniasis is endemic, making them susceptible to the disease. Annually, an estimated 1-2 million new cases are reported, contributing to a global infection prevalence exceeding 12 million people.^1^^,^^2^ Protozoan members of the Leishmania genus are implicated as the causative agents, with Leishmania tropica and Leishmania major being the most encountered species in the Pakistani population.^3^^,^^4^ Clinical manifestations comprise a wide variety of skin lesions, ranging from a traditional oriental sore to a widespread disease with metastatic cutaneous ulcerations commonly seen on exposed body parts such as the face, arms, hands, and feet. Although most lesions are not painful, there is a significant stigma attached to the condition. Factors such as poor sanitation, livestock, and sleeping in open areas confer an increased risk of infection and supposedly explain the increased prevalence among persons of low socioeconomic status.^5^

CL in Pakistan was previously confined to endemic areas, but migration due to wars and geopolitical instability has spread it to nonendemic regions, raising public health concerns. Cases are now emerging in nonendemic areas, with outbreaks in high-burden regions.^6^^,^^7^ Baluchistan (a southwestern province), a predominantly rural area, bears the highest disease burden, with its capital city, Quetta, experiencing particularly high prevalence despite being an urban center. This is followed by Khyber Pakhtunkhwa (KPK; a northwestern province), which includes both rural areas and urban hubs like Peshawar, along with Sindh (a southeastern province) and Punjab (a northeastern province), both of which also have a high prevalence of the disease.^3^^,^^5^ High prevalence and extremely low awareness, particularly in endemic regions, have fueled the spread of CL into urban areas like Karachi, further straining Pakistan's already limited health care system.^6^^,^^8^, ^9^, ^10^

This study aims to describe the epidemiology of CL by documenting the clinical characteristics of cases presenting to our institute and mapping their geographic distribution based on the patients' city of origin. Additionally, it explores the role of travel history to endemic areas among patients originating from Karachi. The study will provide actionable insights for public health officials and policymakers to develop strategies for curtailing the spread of the disease and preventing an endemic situation. Through this collaborative effort, we endeavor to mitigate the burden of CL and contribute to its control at the population level.

## Methodology

### Ethical declaration and informed consent

The Institutional Review Board and Ethics Committee at the Institute of Skin Diseases Sindh Karachi granted ethical clearance (D.O. No. DISDSK 5082), endorsing both data gathering and subsequent analyses, aligning rigorously with the tenets outlined within the Declaration of Helsinki. Detailed verbal and written informed consent are routinely secured for both participation in the research study and the collection of samples for microscopy as part of the institutional protocol. Participants are thoroughly educated about the potential risks and benefits of the procedures involved before deciding to participate in our usual clinical settings.

### Research framework and participants

This investigation was undertaken retrospectively at the Leishmaniasis Clinic of Pakistan’s leading dermatological facility, spanning from July 2019 to February 2024. Serving a large patient population from across Pakistan, individuals presenting with suspected clinical manifestations of CL were assessed using comprehensive diagnostic modalities, including smear microscopy, histopathological examination, and/or serological assays. Only untreated patients with confirmed diagnosis of CL were included in the study. Patients were included if they provided comprehensive clinical history, including travel history and lesion duration. Exclusions were made for cases of visceral or mucocutaneous leishmaniasis, individuals with insufficient information due to recall limitations, and those who did not undergo diagnostic tests after initial assessment. Participants were enrolled using a consecutive sampling approach, and no precalculation of sample size was performed.

The demographic profile, including age, gender, residence, and travel history to endemic areas within the 6 months preceding the appearance of the lesion, was systematically recorded at the time of presentation at the clinic visit. Clinical parameters such as lesion site (exposed or unexposed), duration of lesions, lesion count, and lesion size were also meticulously documented during the initial assessment.

### Sampling and microscopy

Each participant underwent a slit skin smear procedure under sterile conditions. Following the removal of the overlying dry scab with an alcohol-soaked gauze, local anesthetics were administered to alleviate the pain associated with the sampling process. Tissue material was meticulously collected using a scalpel, transferred onto slides, and stained with Giemsa. The slides were then examined under a light microscope at 100× magnification to detect the presence of Leishmania spp. amastigotes, or Leishman-Donovan (LD) bodies. A slide was deemed positive if at least 2 amastigotes or LD bodies were observed. Each case underwent thorough examination by a skilled pathologist. In instances of negative results, patients underwent further evaluation through histopathological examination and serological assays using venous sampling.

### Treatment

Treatment followed the protocols outlined by the WHO.^11^ Patients presenting with fewer than 4 lesions or lesions measuring less than 4 centimeters received intralesional antimonials, whereas those with more than 4 lesions or lesions exceeding 4 centimeters in size received intramuscular antimonials until resolution.

### Statistical analysis

Data for CL were entered and analyzed using Microsoft Excel and SPSS version 22 (IBM Corp.). Percentages were reported for the demographic and clinical characteristics, including age, gender, lesion duration, lesion count, anatomical distribution, travel history, and geographical distribution (by city and province). The chi-square test was used to compare positive travel histories between individuals from Karachi and other regions. The Jonckheere–Terpstra test for trend was applied to compare the distribution of CL cases across months, with data from the same month across different years combined into single groups. Statistical significance set at a *P* value <.05.

## Results

**Fig 1:**
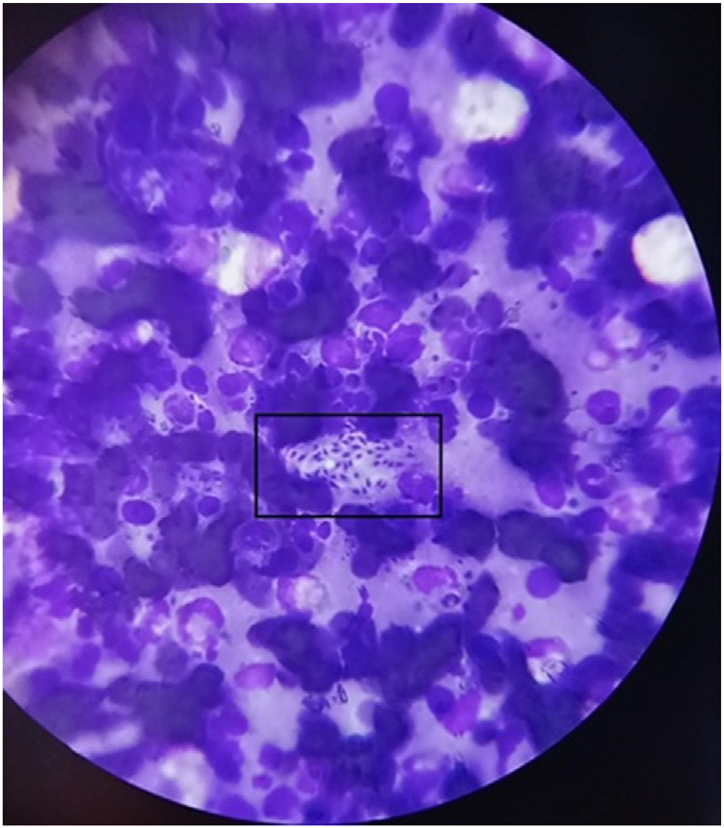
Leishman-Donovan bodies under a light microscope at 100× magnification.

This study analyzed a cohort of 525 individuals, encompassing both male and female participants, over the period from July 2019 to February 2024 (Supplementary Fig 1, available via Mendeley at https://data.mendeley.com/datasets/nptcw7kwdz/1). The evaluation included factors such as age, gender, duration of lesions at presentation, patients’ origin, travel history to endemic areas within the last 6 months, lesion count, lesion size, and the specific anatomical regions, categorized into exposed and unexposed, affected by CL confirmed by microscopy. Fig 1 shows LD bodies under a light microscope at 100× magnification.

### Age and gender demographics of CL distribution

The distribution of CL cases ranged from infancy to 70 years, depicted that younger demographics accounted for a greater proportion of cases. Among the 525 participants, 18.9% were in the 0-5 age bracket, 16.6% were 6-10 years old, with prevalence decreasing in older age groups. Percentages for other age groups are detailed in Table I.

**Table I:** Age and gender distribution of cutaneous leishmaniasis

Age	*N* (%)
0-5	99 (18.9)
6-10	87 (16.6)
11-15	53 (10.1)
16-20	47 (9.0)
21-25	55 (10.5)
26-30	28 (5.4)
31-35	34 (6.5)
36-40	32 (6.1)
41-45	21 (4.0)
46-50	28 (5.4)
51-55	17 (3.2)
56-60	10 (1.8)
61-65	10 (1.8)
66-70	4 (0.7)
Gender	
Male	342 (65.1)
Female	183 (34.9)

In terms of gender distribution, males are more commonly affected, comprising 65.1% (342 individuals) of the cases, compared to 34.9% (183 individuals) for females (Table I).

### Lesion duration at presentation

CL lesions varied in duration, from less than a month to more than 5 years. Among the 525 participants, 56.6% had lesions for 0-4 months, 26.7% for 5-8 months, and 11.6% for 9-12 months (Supplementary Table I, available via Mendeley at https://data.mendeley.com/datasets/nptcw7kwdz/1). Monthly case numbers showed no significant differences (*P* = .49) (Supplementary Fig 2, available via Mendeley at https://data.mendeley.com/datasets/nptcw7kwdz/1).

### Anatomical distribution of lesions

**Fig 2:**
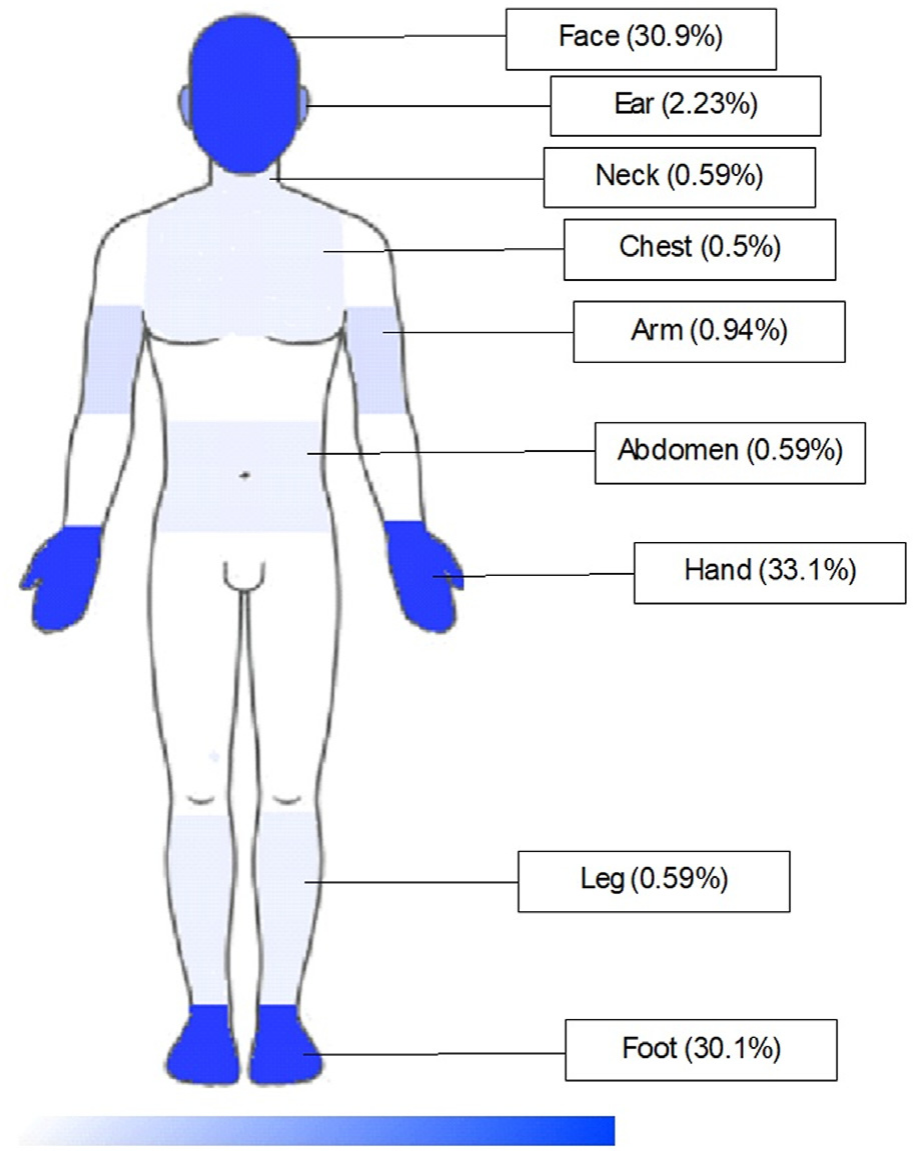
Body map illustrating the affected areas.

**Fig 3:**
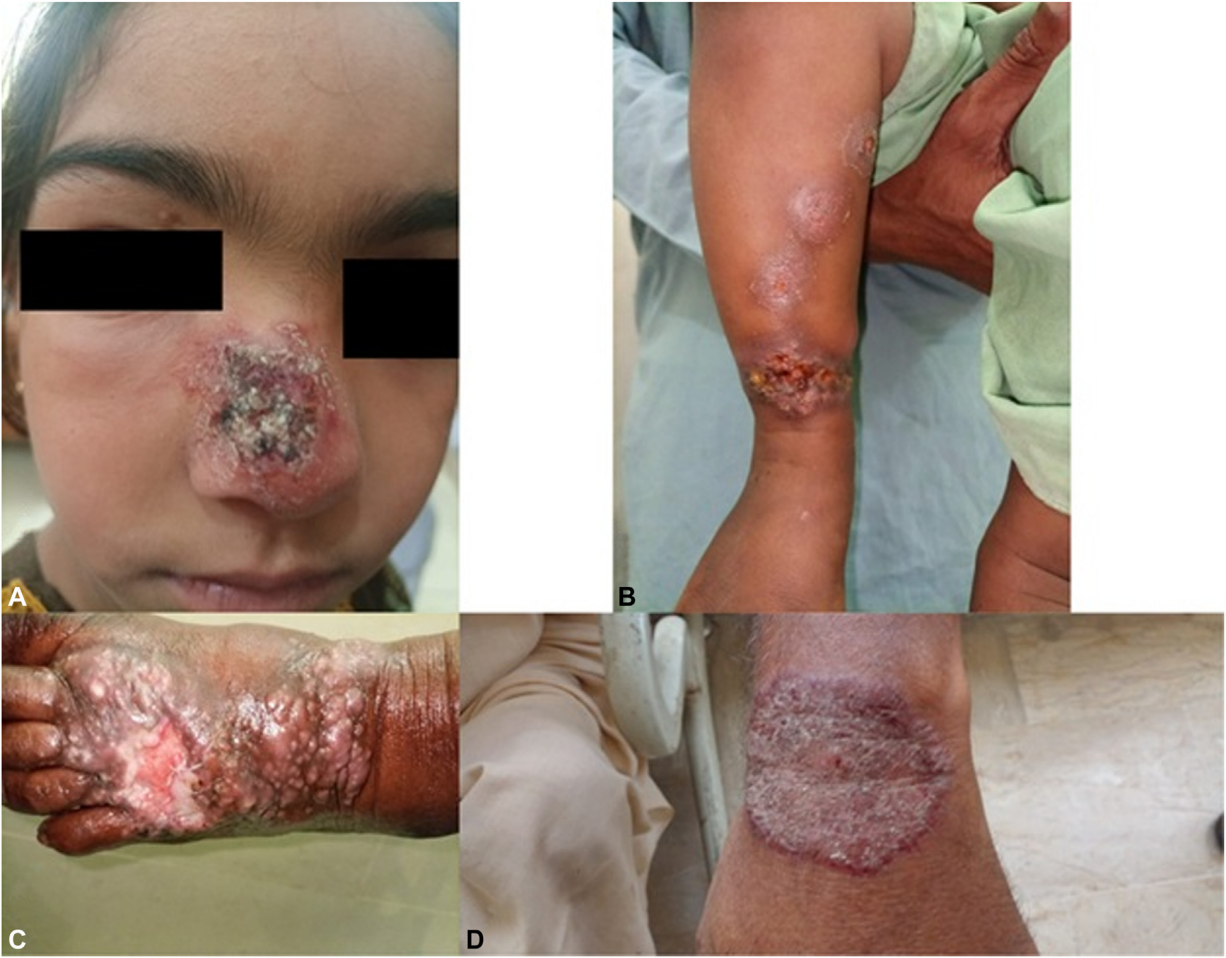
Cutaneous leishmaniasis (CL) lesions observed across different body regions (**A**) face, (**B**) leg, (**C**) foot, (**D**) hand.

CL lesions predominantly affected exposed areas of the body, accounting for 96.3% of cases: 33.1% on the hands, 30.9% on the face, 30.1% on the feet, and 2.3% on the ear. The remaining 3.7% were located on unexposed regions, such as the legs and chest (Supplementary Table II, available via Mendeley at https://data.mendeley.com/datasets/nptcw7kwdz/1). The body map illustrating the affected areas is presented in Fig 2. Lesion counts per individual ranged from 1 to 7, with sizes varying between 1 cm and 15 cm. Approximately 64.2% had a single lesion, 19.6% had 2, and 1 patient had 7 lesions. Detailed information is provided in Supplementary Table III, available via Mendeley at https://data.mendeley.com/datasets/nptcw7kwdz/1. CL lesions observed across different body regions are depicted in Fig 3.

### Patient residence

#### By province

CL cases show regional variation. Patients coming from Baluchistan accounted for the largest proportion of cases (49.7%), followed by Sindh (26.1%) and KPK (21.3%).

#### By city

At our institute, many cases reported were from Quetta (23.2%) and Karachi (21%). Additional cases were identified from regions such as Lasbella, Waziristan, Peshawar, and Hub Chowki, while fewer cases were reported from Kohat, Mansehra, and Mohmand Agency. Cases were also observed from various other regions, reflecting the widespread presence of the disease (Table II).

**Table II:** City and province of residence of cutaneous leishmaniasis patients

City of residence	*N* (%)
Quetta	122 (23.2)
Karachi	110 (21)
Peshawar	33 (6.3)
Lasbella	32 (6.1)
Waziristan	29 (5.5)
Hub Chowki	24 (4.6)
Kohat	17 (3.2)
Mansehra	16 (3.0)
Mohmand Agency	14 (2.7)
Windar	13 (2.5)
Others	115 (21.9)
Province of residence	
Baluchistan	261 (49.7)
Sindh	137 (26.1)
KPK	112 (21.3)
Punjab	15 (2.9)

*KPK*, Khyber Pakhtunkhwa.

### Travel history to endemic areas

Among the 110 cases reported from Karachi, 41 (37.3%) had a travel history to endemic areas, whereas only 9 out of 415 (2.17%) cases from other regions reported travel to endemic areas. These findings were statistically significant (*P* < .001), indicating distinct transmission patterns in Karachi. Additionally, 69 patients (62.7%) from Karachi reported no travel history but still developed CL, suggesting the possibility of local transmission (Table III). The regions to which patients from Karachi traveled are listed in Supplementary Table IV, available via Mendeley at https://data.mendeley.com/datasets/nptcw7kwdz/1.

**Table III:** Travel patterns among cutaneous leishmaniasis patients from Karachi versus other cities

Travel history	Live in Karachi	Other cities	*P* value
Traveled	41	9	
Did not travel	69	406	<.001
Total	110	415	

## Discussion

The prevalence and geographic distribution of CL have significantly expanded in Pakistan, with endemic areas, especially in regions with Afghan refugees in Baluchistan, KPK, Sindh, and scattered occurrences in Punjab.^12^ Additionally, as a tropical nation in the northwestern region of South Asia, sharing borders with leishmaniasis-endemic countries such as China, Afghanistan, Iran, and India, contributes to this issue.^13^ CL is of clinical importance due to its chronicity and its potential for local destruction and disfigurement if healed without medical intervention, making it a substantial public health concern.^14^ Numerous studies have extensively documented the prevalence and distribution of CL across various regions of Pakistan.^13^^,^^15^, ^16^, ^17^, ^18^, ^19^, ^20^, ^21^, ^22^

This study was conducted at an institute situated in Karachi, a coastal city at sea level that shares a border with Baluchistan, where a study found over 4000 cases of CL from August 2018 to December 2019.^18^ The initiation of this study was prompted by numerous CL cases reported to our institute in recent years. Furthermore, there is a lack of data on CL cases in Karachi, prompting investigation into whether Karachi is experiencing endemicity or if the increase in patient numbers at our institute is due to individuals coming from endemic areas or with significant travel histories. Therefore, this study aims to comprehensively investigate epidemiology and assess the clinical presentations of CL cases observed at our facility.

CL infection exhibits a higher prevalence among males compared to females, consistent with similar findings reported by Iqbal et al,^19^ Hussain et al,^23^ Saliba et al,^24^ and Iddawela et al.^25^ The higher incidence of CL in males is linked to their frequent outdoor activities and close contact with animals due to low socioeconomic status, which increases their risk of sand fly bites. Additionally, sleeping on the ground further enhances their susceptibility. In contrast, females, who primarily stay indoors, have reduced exposure to these vectors.^19^^,^^26^ The current study indicated that 54.6% of CL cases were reported in individuals aged 0-20 years. This finding aligns with other research conducted in various regions of Pakistan, including studies by Shaheen et al (2020) in Azad Jammu and Kashmir,^27^ in KPK by Ullah et al (2024),^28^ and by Arif M et al (2022) in Bajaur.^22^ International studies also support these results, such as those by Yohannes M et al (2019) in Ethiopia,^29^ Aara et al (2013) in India,^30^ and Khosrotaj et al (2022) in Iran.^31^

This increased susceptibility in children may be attributed to compromised immune systems and malnutrition, including poor diet. Poverty and limited healthcare further heighten their vulnerability, leading to higher CL incidence in younger populations.^32^ We observed a decline in CL cases with age. This trend aligns with existing literature, which shows a decline in CL cases with age, as adults typically develop lifelong immunity after initial infection.^18^^,^^20^^,^^21^^,^^27^

Our study uncovered that CL lesions primarily appear on exposed body areas. Additionally, approximately 64.2% of participants had a single lesion, while 19.6% presented with 2 lesions. Comparable findings were noted in studies conducted in Baluchistan,^18^ Multan,^15^ KPK.^28^ CL lesions often manifest on exposed areas due to their typical lack of coverage, particularly during sleep, which facilitates sand flies transmitting the parasite to these areas. CL cases were documented throughout the year, with peaks observed in January, February, September, March, October, and November. Conversely, April and May showed the lowest incidence of cases. However, contrary to this observation, literature indicates that the peak season for CL in Pakistan occurs during the warm months. This pattern is likely due to the heightened activity of sand flies in warmer weather, which necessitates more blood for their egg development.^18^^,^^27^ The variation between our study and the literature may be attributed to the duration of lesion presentation at our institute, ranging from months to 5 years.

CL cases show significant regional disparities, with Baluchistan and Sindh having the highest incidence. Quetta and Karachi were the most affected areas at our institute. Notably, 37.3% of Karachi cases had a travel history, whereas only 2.2% of cases from outside Karachi reported travel, with a significant *P* value of < .001. This highlights the impact of travel on CL transmission in urban settings like Karachi. CL is endemic to Baluchistan, KPK, and certain areas in Sindh and Punjab, particularly those adjacent to the Baluchistan border.^13^^,^^15^, ^16^, ^17^, ^18^^,^^20^, ^21^, ^22^^,^^28^^,^^33^ Historically, Karachi, the most populous city in the country, was not endemic for CL. However, in recent years, multiple cases have emerged, with several studies attributing this surge to travel history or migration from endemic areas within Pakistan.^7^^,^^13^^,^^17^^,^^34^^,^^35^ Alarmingly, 67% of Karachi patients in this study had no travel history yet still developed the disease, indicating potential local transmission factors and highlighting an emerging public health concern in a city that was previously nonendemic.

## Suggestions

CL prevention and control strategies are crucial for curbing the spread of this parasitic disease. Effective measures include managing sand fly habitats by clearing vegetation, improving waste disposal, and addressing stagnant water. Vector control through indoor residual spraying and insecticide-treated nets is vital. Community engagement to raise awareness of CL symptoms and promote preventive behaviors, such as using repellents and protective clothing, is essential. The finding that 94% of patients had lesions on exposed sites underscores the need for preventive measures like covering skin and using protection.

Robust surveillance systems are crucial for monitoring outbreaks and enabling timely interventions. Health authorities should strengthen case detection, train healthcare personnel, and ensure access to diagnostics and treatments. To prevent CL from spreading to non-endemic areas, implementing rigorous advisories and screening for travelers, migrants, and livestock from high-burden regions like Baluchistan is essential. Coordinating with local authorities to enhance preventive measures is critical, given that approximately 50% of CL cases originate from Baluchistan.

We recommend that Pakistan be included in the leishmaniasis control vision plan for 2030 and stress the urgent need for new drug development and treatment modalities. Immediate and coordinated action is necessary, including integrating Pakistan into the WHO's leishmaniasis eradication plan.

## Conclusion

This study highlights the prevalence of CL in Karachi, Pakistan, and emphasizes the role of travel history to endemic areas in the disease transmission dynamics, particularly in urban settings such as Karachi. It further stresses the urgent need for enhanced surveillance, prevention, and treatment strategies.

## Conflicts of interest

None disclosed.
